# Association between inflammatory bowel disease and pancreatic cancer: results from the two-sample Mendelian randomization study

**DOI:** 10.3389/fonc.2023.1155123

**Published:** 2023-08-23

**Authors:** Yu Min, Zheran Liu, Ruidan Li, Jing Jin, Zhigong Wei, Yiyan Pei, Xiaolin Hu, Xingchen Peng

**Affiliations:** ^1^ Department of Biotherapy and National Clinical Research Center for Geriatrics, Cancer Center, West China Hospital, Sichuan University, Cheng Du, Sichuan, China; ^2^ West China School of Nursing, West China Hospital, Sichuan University, Cheng Du, Sichuan, China

**Keywords:** inflammatory bowel disease, Crohn’s disease, ulcerative colitis, risk factor, pancreatic cancer, mendelian randomization, GWAS

## Abstract

**Background:**

The nuanced relationship between inflammatory bowel disease (IBD) and pancreatic cancer is noticed in recent years. However, the underlying causal effects of these two diseases are still unclear.

**Methods:**

The two-sample mendelian randomization (MR) was conducted to explore the causal effect of IBD condition on pancreatic cancer. Methods of Wald ratio, inverse variance weighted (IVW), MR-Egger, weighted median, and weighted mode were used to investigate the causal relationship between IBD and pancreatic cancer. Besides, Cochrane’s Q test, MR-Egger, and leave-one-out method were further conducted to detect heterogeneity, stability, and pleiotropy of MR results.

**Results:**

In the MR analysis, we found Crohn’s disease had a significant causal effect on pancreatic cancer. Specifically, Crohn’s disease would increase 11.1% the risk of pancreatic cancer by the IVW method (p= 0.022), 33.8% by MR Egger (p= 0.015), by 35.3% by the Weighted model (p= 0.005). Regarding ulcerative colitis, there was no statistically significant causal effect observed on pancreatic cancer (p>0.05). Additionally, the pleiotropic test and Leave-one-out analysis both proved the validity and reliability of the present two-sample MR analyses.

**Conclusion:**

This study indicates that IBD, particularly Crohn’s disease, is causality associated with increased risk of pancreatic cancer. Our results may help public health managers to make better follow-up surveillance of IBD patients.

## Introduction

The prevalence of inflammatory bowel disease (IBD), classified as Crohn’s disease (CD) and ulcerative colitis (UC), is increased substantially in many regions during the past decades, especially in Western societies such as Europe and North America (1, 2). Results of the IBD statistical report, in 2017, there were 6.8 million cases of IBD worldwide. The age-standardized prevalence rate increased from 79.5 per 100,000 population in 1990 to 84.3 per 100,000 population in 2017 (1). As one typical chronic inflammatory disease of the gastrointestinal tract, an increased cancer risk has also been determined in IBD patients during long-term follow-up (3, 4). Also, IBD treatments, such as immunosuppressant medications, may independently increase the risk of cancer in IBD (5–9).

In recent years, numerous studies were settled to assess the association between IBD and varied cancer risks. Compelling evidence has proved the positive association between IBD and future increased cancer risks. Especially, colorectal cancer is now a well-known risk in IBD patients during follow-up (3, 10–12). Moreover, with a deeper understanding of this chronic inflammation disease, relationships between extraintestinal cancers and IBD also raised clinicians’ interest and concerns (13). For instance, in one prospective observational cohort study within almost half a million populations in European, Wu et al. determined an increased risk of digestive, non-melanoma skin cancer, and male genital cancers in IBD patients (14). Similarly, results from Korean (7) and Chinese (4) cohorts also noticed a higher cancer risk in IBD patients, when compared with the background population.

Moreover, as one of the leading causes of cancer mortality globally with increasing prevalence and mortality, pancreatic cancer is considered to have an unfavorable clinical outcome (15, 16). Nevertheless, pancreatic cancer was frequently mixed with other upper gastrointestinal cancers during the calculations (14, 17, 18). To date, limited studies have evaluated the relationship between IBD and pancreatic cancer (7, 19). Notably, Everhov et al. conducted a large-scale population-based study to explore the association between IBD and pancreatic cancer. The results revealed that the 20-year cumulative incidence was 0.34% (95%CI: 0.30-0.38) vs 0.29% (95%CI: 0.28-0.30) and the overall hazard ratio (HR) for pancreatic cancer was increased overall in IBD population (Crohn’s disease: HR= 1.44; ulcerative colitis: HR=1.35, respectively) (19). However, a recent meta-analysis did not find any significant difference in the development of pancreatic cancer among the IBD population and the non-IBD population (20). Some scholars even assumed that the increased pancreatic risk might be due to misdiagnosis of periampullary cancer (13), as the IBD patients with primary sclerosing cholangitis had remarkably higher pancreatic cancer risk (HR=7.55) (19).

There is still a lack of robust evidence to conclude the association between IBD and pancreatic cancer, as previous studies were mainly based on clinical cohort data. Although some potential associations between IBD and pancreatic cancer were suggested, none of them could explain the exact causal relationship. Notably, gene prediction based on genome-wide association study (GWAS) data seems to be able to help us solve this puzzle. The application of Mendelian randomization (MR) methods (21–24) can assist us in predicting causality at the genetic level by correlating GWAS data of IBD and pancreatic cancer. The findings from MR analysis might provide us with insightful perspectives to deeply understand the genetic association between IBD and pancreatic cancer.

To fill this research gap, we aim to investigate the causal effect of IBD and pancreatic cancer, which can provide more robust evidence for clinical IBD management and help the active surveillance, early diagnosis, and precise clinical decision for high-risk pancreatic cancer subpopulation.

## Materials and methods

### Data source

The classification of IBD incorporates two sub-diseases, named ulcerative colitis and Crohn’s disease. The GWAS data for IBD in this study was derived from the International Inflammatory Bowel Disease Genetics Consortium (IIBDGC, an organization aimed at identifying genetic risk factors for IBD and their clinical features as well as assessing the underlying interaction between genetic risks and the disease phenotypes). Specifically, the GWAS data of ulcerative colitis (GWAS id: ieu-a-970) was derived from the summary data of Liu et al. (25) and further download from the “MR-base”, a platform for MR developed by the “MRC Integrative Epidemiology Unit” at the University of Bristol (26) (https://gwas.mrcieu.ac.uk/datasets/ieu-a-970/). It contained 13,768 European-descent IBD cases and 33,977 European-descent health control with 156,116 single-nucleotide polymorphisms (SNPs). Similarly, the GWAS data for Crohn’s disease (GWAS id: ieu-a-12) was also extracted from the summary data of Liu et al. (25) and further download from the “MR-base” (https://gwas.mrcieu.ac.uk/datasets/ieu-a-12/). The GWAS data of Crohn’s disease contained 17,897 European-descent IBD cases and 33,977 European-descent health control with 124,888 SNPs. The data on pancreatic cancer was primarily derived from the Pancreatic Cancer Cohort Consortium release 1 (PanScan 1), including a large-scale GWAS analysis of 3,835 people from 12 prospective cohorts plus one hospital-based case-control study (27), and we downloaded the GWAS data from the same platform (https://gwas.mrcieu.ac.uk/datasets/ieu-a-822/). Ulcerative colitis and Crohn’s disease were defined as the exposure factor and pancreatic cancer was determined as the primary outcome.

### SNPs selection and assumption

Our study satisfied the assumptions of MR analysis. First and foremost, the SNPs selected for MR analysis must be strongly associated with ulcerative colitis and Crohn’s disease. In the present study, to ensure satisfy the assumption, only SNPs in IBD whose p-values were below the genome-wide significance level (5 × 10^−8^) were included for analysis. To ensure the robust association between instrumental variables and exposure factors, we excluded the weak instrumental variables with F values (formula: (R^2^/(R^2^-1)) *((N-K-1)/K)) <10 (28). Detailly, N represents the sample size of the exposure dataset, K is the number of SNPs, and R^2^ is the proportion of variation explained by IVs in the exposure dataset. Secondly, the chosen instrumental variables must meet an independence test. Genetic distance refers to the length of the region, regarding the linkage disequilibrium. Therefore, 1000 Genomes project European samples data were used as the reference panel to calculate the linkage disequilibrium between the SNPs (29). Namely, the SNP linkage disequilibrium value (*r* ^2^) was set to 0.001 and the genetic distance was set to 10000 kb. We removed the SNP with *r* ^2^ greater than 0.001 with the most significant SNPs to reduce the linkage disequilibrium impact and keep the independence of selected instrument variables. The SNPs characteristics of IBD from the European population were collected, including the number of SNPs, chromosome location, effective allele, effective allele frequency, effect value, standard error, and associated P-value of the effective allele.

**Figure 1:**
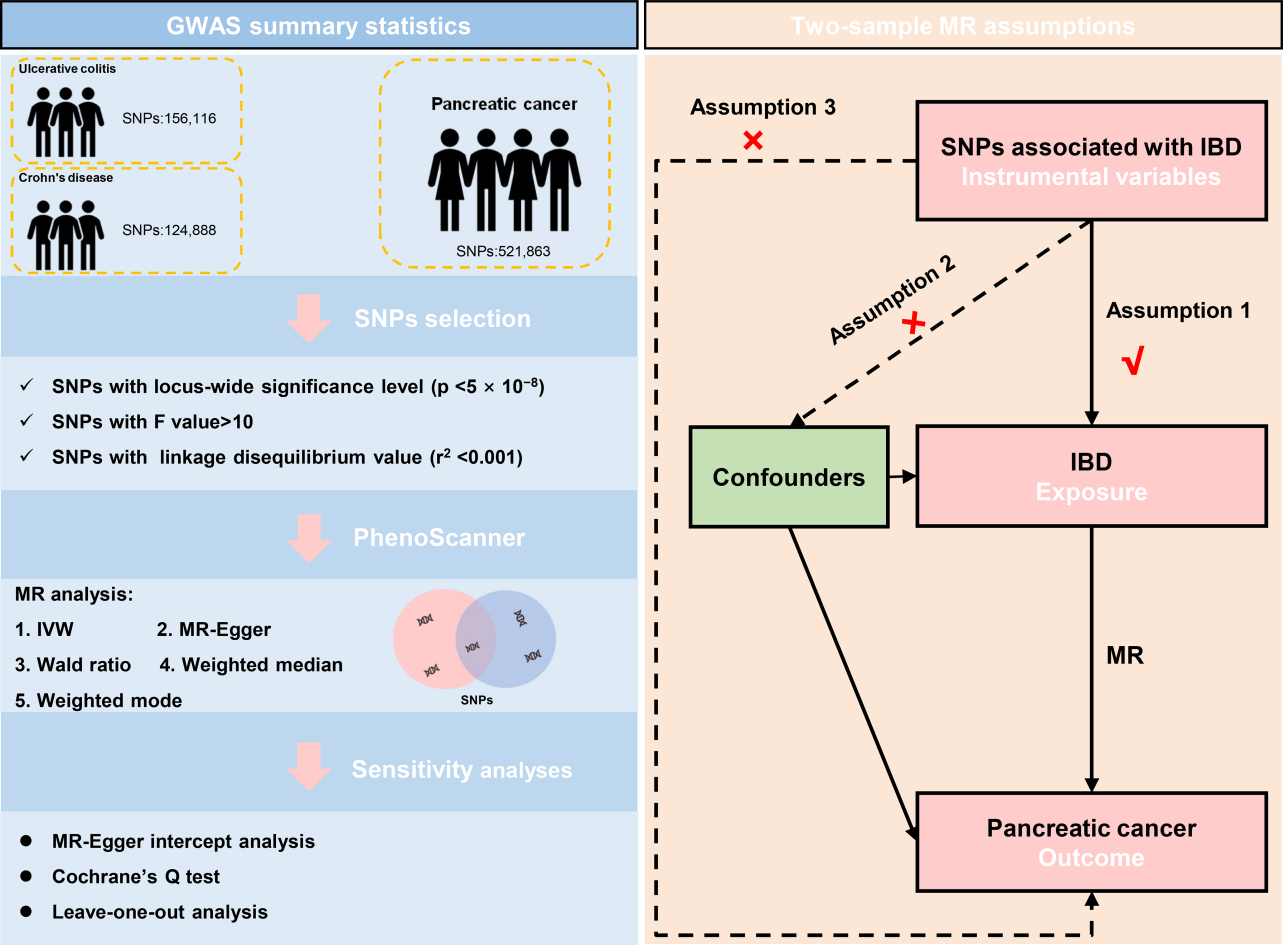
The data selection and the assumptions for the Mendelian randomization analysis in the present study. SNP, single-nucleotide polymorphism; IVW, inverse variance weighted; IBD, inflammatory bowel disease; MR, Mendelian randomization.

Besides, Each SNP was checked at PhenoScanner V2 (30), which was a database of human genotype-phenotype associations (http://www.phenoscanner.medschl.cam.ac.uk/). Based on the PhenoScanner, the SNPs significantly associated with the potential confounders, like significantly existing in other tissues or diseases, in the present study were subsequently excluded. The mentioned instrumental variables screening guarantees the validity of our study’s findings. The schematic diagram for the Mendelian randomization analysis was presented (**Figure 1**).

### Two-sample Mendelian randomization analysis

Four methods, including Inverse variance weighting (IVW), MR-Egger, weighted median, and weighted mode, were performed to assess the causal effect between exposures and outcomes. Different methods have different sensitivities to various issues, accommodate different scenarios, and vary in their statistical efficiency. As the primary analysis method, IVW is a time-honored method for combining the Wald ratio estimates of all relevant instrumental variables. This strategy is analogous to using weighted linear regression to probe the ties between the instrumental factors and the result. The instrumental variables’ intercept is restricted to zero. IVW can obtain unbiased estimates of the status without horizontal pleiotropy. Under the premise of Instrument Strength Independent of Direct Effect (InSIDE) (31), the MR-Egger method can primarily demonstrate the dosage relationship between instrumental variables and outcomes while accounting for some pleiotropy. The class one error rate can be lowered using the weighted median method, which also permits the possibility of invalidity for some specific genetic variants. Even if a certain instrumental variable does not satisfy the requirements of the MR technique for causal inference, the weighted mode approach remains valid when the vast majority of instrumental variables with identical causal estimates are valid. If the results of these methods are inconsistent, we give priority to IVW as the main result.

Furthermore, pleiotropy (refers to a genetic variant with numerous independent phenotypic effects), which might affect the causal effects, was assessed by the method of MR Egger intercept. To verify the conformity of each SNP, the heterogeneity test was performed utilizing MR Egger and IVW methods to calculate Cochran Q statistics and find the heterogeneity among genetic variants (32). Meanwhile, the leave-one-out analysis was conducted by excluding the genetic variants one by one. Then, the causal relationship would be rational and stable if the result of the leave-one-out analysis was in line with the global IVW analysis. To determine a more rigorous interpretation of the causal relationship between IBD and pancreatic cancer, Bonferroni-corrected was used in this study, according to the number of study exposures (0.05/2, 0.025). A nominal causal effect was determined when the p-value was between 0.05 and the corrected value of 0.025.

### Statistical analysis

This study was followed by the STROBE-MR guideline for designation (33). All of the statistical analyses were performed by R (version 4.2.0, https://www.rproject.org/) with the “TwoSampleMR” package.

## Results

### Selected GWASs of IBD

From the same European ancestry, there were 103454 cases involved in the present study, including 47,745 cases (13,768 patients and 33,977 health control) with 156,116 SNPs in the ulcerative colitis group, 51,874 cases (17,897 patients and 33,977 health control) with 124,888 SNPs in Crohn’s disease group, and 3835 cases (1,896 patients and 1,939 health control) with 521,863 SNPs in pancreatic cancer group, respectively. Moreover, there were 80 instrumental variables in ulcerative colitis and 122 instrumental variables in Crohn’s disease selected to conduct the MR analysis (**Table 1**). The detailed information of each selected SNP was summarized in Supplementary Files (**Supplementary Files**).

**Table 1:** The list of Genome-wide summary association studies (GWAS) included in the Mendelian randomization (MR) study.

Disease	GWAS ID	Sex	Sample size	Cases	Control	No. SNPs	Consortium	Population
Ulcerative colitis	ieu-a-970	F/M	47,745	13,768	33,977	156,116	IIBDGC	European
Crohn’s disease	ieu-a-12	F/M	51,874	17,897	33,977	124,888	IIBDGC	European
Pancreatic cancer	ieu-a-822	F/M	3835	1,896	1,939	521,863	PanScan 1	European

F/M, female and male; SNPs, single-nucleotide polymorphisms; PanScan 1, Pancreatic Cancer Cohort Consortium released 1.

### Two-sample Mendelian randomization analysis

**Figure 2:**
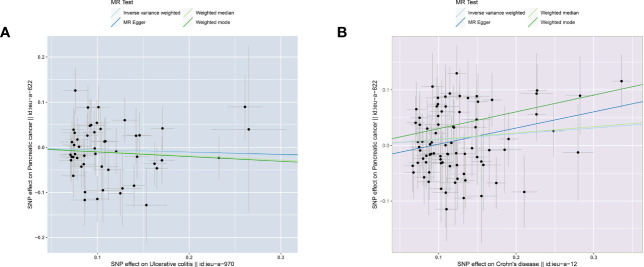
Scatter plots of causal estimates from genetically predicted IBD on pancreatic cancer. **(A)** ulcerative colitis. **(B)** Crohn’s disease.

The two-sample MR analysis was conducted to investigate the causal effect of IBD on pancreatic cancer. Specifically, ulcerative colitis was not observed to have causal effect on pancreatic cancer (**Figure 2A**) across the IVW (OR= 0.946, 95% CI= 0.830-1.079, p= 0.409), Weighted median (OR= 0.910, 95% CI= 0.753-1.101, p= 0.332), Weighted mode (OR= 0.902, 95% CI= 0.682-1.194, p= 0.474), and MR-Egger (OR= 0.951, 95% CI= 0.648-1.397, p= 0.799) methods (**Table 2**). Alternatively, there was a significant causality between Crohn’s disease and pancreatic cancer, namely, Crohn’s disease would increase the risk of pancreatic cancer (**Figure 2B**).

**Table 2:** Two sample MR analysis results of ulcerative colitis and pancreatic cancer.

Exposure	MR Method	No. SNPs	β	SE_β_	OR (95%CI)	*P*
**Ulcerative colitis**	MR Egger	51	-0.050	0.196	0.951(0.648-1.397)	0.799
	Weighted median		-0.094	0.097	0.910(0.753-1.101)	0.332
	Inverse variance weighted		-0.055	0.067	0.946(0.830-1.079)	0.409
	Weighted mode		-0.103	0.143	0.902(0.682-1.194)	0.474

MR, Mendelian randomization; SNPs, single-nucleotide polymorphisms; SE, standard error; OR, odd ratio.

**Figure 3:**
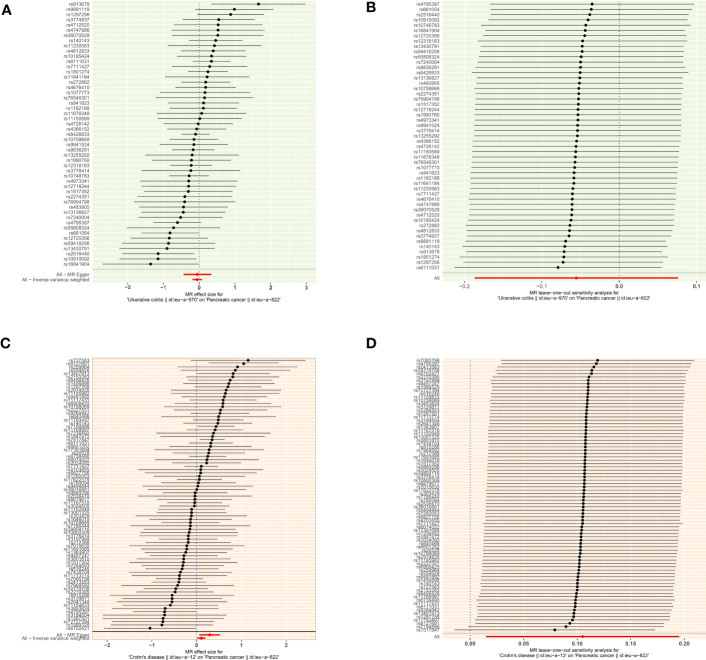
The causal effect of exposure on the outcome and leave-one-out sensitivity analysis for each SNP **(A)** The causal effect of exposure on the outcome is estimated using each SNP for ulcerative colitis and pancreatic cancer. **(B)** Leave-one-out sensitivity analysis for each SNP for ulcerative colitis and pancreatic cancer. **(C)** The causal effect of exposure on the outcome is estimated using each SNP for ulcerative colitis and pancreatic cancer. **(D)** Leave-one-out sensitivity analysis for each SNP for Crohn’s disease and pancreatic cancer.

According to the MR analysis, Crohn’s disease could increase approximately 11.1% risk of pancreatic cancer by the IVW method (OR= 1.111, 95% CI= 1.015-1.213, p= 0.022), by 33.8% by MR Egger (OR= 1.338, 95% CI= 1.064-1.683, p= 0.015), by 35.3% by the Weighted model (OR= 1.353, 95% CI =1.100-1.662, p= 0.005) methods (**Table 3**). Each SNP singly estimated the causal effect of IBD on pancreatic cancer by using the Wald ratio method, which was represented in two forest plots (**Figures 3A, C**). Moreover, the leave-one-out method revealed that the MR results remained stable after excluding the instrumental variables one by one. (**Figures 3B, D**).

**Table 3:** Two sample MR analysis results of Crohn’s disease and pancreatic cancer.

Exposure	MR Method	No. SNPs	β	SE	OR (95%CI)	*P*
**Crohn’s disease**	MR Egger	82	0.291	0.117	1.338(1.064-1.683)	**0.015**
	Weighted median		0.113	0.073	1.120(0.970-1.292)	0.119
	Inverse variance weighted		0.105	0.046	1.111(1.015-1.213)	**0.022**
	Weighted mode		0.302	0.105	1.353(1.100-1.662)	**0.005**

MR, Mendelian randomization; SNPs, single-nucleotide polymorphisms; SE, standard error; OR, odd ratio.

Bold values indicate statistical significance (p<0.05).

### Horizontal, sensitivity analysis, and Bonferroni-corrected test

**Figure 4:**
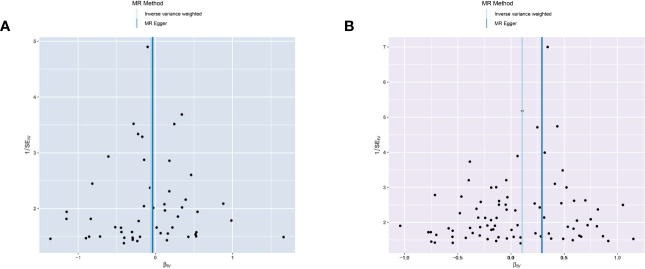
Funnel plot to assess the heterogeneity in causal estimates from genetically predicted IBD on pancreatic cancer. **(A)** ulcerative colitis. **(B)** Crohn’s disease.

The MR-Egger analysis showed that there was no horizontal pleiotropy or outliers in MR analysis for IBD and pancreatic cancer (Egger intercept= -0.001, Standard error= 0.022, p= 0.978 in ulcerative colitis, and Egger intercept= -0.026, Standard error= 0.015, p= 0.087 in Crohn’s disease, respectively). The funnel plot was adopted to show the distribution balance of single SNP effects (**Figures 4A, B**).

The plots indicated that the effect of each SNP and distribution were in equilibrium. Additionally, to evaluate the heterogeneity in MR analysis, Cochrane’s Q test was also applied. In Crohn’s disease and pancreatic cancer group, the Q was 82 in MR Egger and 85 in inverse variance weighted, respectively (p>0.05). Similarly, in the ulcerative colitis and pancreatic cancer group, the Q was 54 in MR Egger and 54 in inverse variance weighted, respectively (p>0.05). Therefore, the heterogeneity test found limited evidence of heterogeneity. Only Crohn’s disease was observed to achieve a significant causal effect after using the Bonferroni-corrected test.’

## Discussion

To the best of our knowledge, we conducted the preliminary MR analysis on evaluating the causality between IBD and pancreatic cancer in the European population, based on the large-scale GWAS databases. We identified the positive association between Crohn’s disease and pancreatic cancer. The findings might provide noteworthy value for future preclinical studies on this topic.

To date, the association between IBD and pancreatic cancer raised wide concerns (19, 34). Notably, evidence from the Scandinavian register-based cohort study, Everhov et al. determined a statistically significant increased risk of pancreatic cancer in IBD patients. Interestingly, the long-term cumulative incidence was similar between the case and control groups (0.34% vs. 0.29%). Meanwhile, results from a Korean study confirmed an increased pancreatic cancer risk (OR=8.6, 95%CI: 1.0-31.0) in women with Crohn’s disease (7). Herein, some scholars even suggested that patients should not be nervous about this potential risk, while the primary sclerosing cholangitis or misdiagnosis of periampullary cancer would be contributed to this increased risk instead (13, 35). Nonetheless, the exact causal effect of IBD and pancreatic cancer remains unclear and could not be evaluated by observational studies, as the confounders could not be comprehensively adjusted.

In the present study, we noticed that Crohn’s disease but not Ulcerative colitis could significantly increase the risk of pancreatic cancer (IVW analysis: OR= 1.111, p=0.022) via the MR analysis. This divergence was consistent with recent clinical observational studies in different ethnic populations (7, 36). Apart from the IVW analysis, the other two MR analysis methods, including MR Egger (OR=1.338, p=0.015) and Weighted mode (OR= 1.353, p=0.005), also supported that Crohn’s disease caused by genetic factors can elevate the risk of pancreatic cancer. Reviewing the previous study on evaluating Crohn’s disease and pancreatic cancer, the majority of studies yielded that Crohn’s disease could increase pancreatic cancer risk. Focused on the cancer risk in Crohn’s disease patients with more detailed cancer sites, Hemminki et al. found an increased risk for many subsequent cancers, including pancreatic cancer. Interestingly, the highest standardized incidence ratio (SIR) frequently appeared in patients with more active surveillance (Follow-up interval<1 year, SIR=6.15) and elderly patients (>64 years, SIR=3.3). Most recently, in one large population-based study from the UK Biobank prospective database, Wu et al. demonstrated IBD patients had a higher incidence of digestive cancer and worse cancer-specific mortality, compared with reference controls. Moreover, the prevalence of each site-specific cancer was higher in ulcerative colitis patients than that in Crohn’s disease patients. As for pancreatic cancer, however, it was included in “Other digestive cancers” during analysis and there was no remarkable increased cancer risk observed in the results, after adjusting for thirteen confounders (14). In our study, no significant increased pancreatic cancer risk was determined in ulcerative colitis patients. Although previous studies also discovered this discrepancy (7, 19), we could not conclude this phenomenon as ulcerative colitis patients showed higher incidence in the majority of cancer sites. Therefore, with more comprehensive genetic data or adjusting more confounders, future works could be settled to further investigate the causality between ulcerative colitis and cancer sites that present relatively lower incidence in the background population.

Currently, the indications of screening for pancreatic cancer are restricted and not recommended for the general population of asymptomatic adults (37). Only when individuals who have a family history of pancreatic cancer, have a pathogenic variant in one of the high-risk pancreatic cancer susceptibility genes, or individuals at high risk owing to a personal history of pancreatic disease might consider screening. Thus, there is a lack of evidence for applying pancreatic cancer screening for individuals with comorbidities like diabetes mellitus, and chronic pancreatitis, even though these diseases are clinically observed to be associated with the risk of pancreatic cancer. Therefore, whether physicians need to inform patients about the increased risk of pancreatic cancer and subsequent annual screening is still a controversial topic (35). With the continually updated GWAS data, the common variants in several genomic regions as significantly associated with pancreatic cancer risk could be identified (16, 27). This milestone could help the development of pancreatic cancer screening with high-accuracy and non-invasive strategies.

There are some strengths of the present study that need to be mentioned. First, we evaluated the causality between IBD and pancreatic cancer via different MR methods. Besides, the design and SNPs evaluation of the study is followed by the guidelines of MR analysis (22). The evidence of MR sits at the interface of experimental and observational studies, which could provide more robust evidence for investigating the causality between two events. Thus, with the heterogeneity and horizontal pleiotropy-sensitive analysis, the findings drawn in this study were reasonable.

Indeed, there are still some limitations that need to be addressed in the following works. First, while the study population in this work eliminates the race discrepancy, whether the findings could be generalized to other countries with different races remain unclear. Thus, GWAS studies from different regions could provide a more comprehensive analysis of the association between IBD and pancreatic cancer. Second, as the IBD patients were collected from different medical centers, the differences in diagnosis methods, information acquisition, and data processing could lead to calculation bias. Third, the GWAS data of outcome was solely derived from the PanScan 1 project, which is the pancreatic cancer data that we can obtain to the greatest extent. If possible, we will continue exploring other pancreatic cancer GWAS datasets to understand the relationship between IBD and pancreatic cancer risk fully. Future data with more SNPs included for these diseases could help validate the results we determined.

## Conclusion

In summary, based on the large-scale MR analysis, our results revealed Crohn’s disease instead of ulcerative colitis had a positive causal effect on pancreatic cancer. Future clinical guidelines and recommendations could consider to make better follow-up management and screening of IBD patients and minimize the risk of secondary malignancies, especially in terms of pancreatic cancer.

## Code availability

The R packages mentioned in the manuscript are available and can be downloaded on the website (https://github.com/MRCIEU/TwoSampleMR). Besides, the code and data for conducting this study have been uploaded on the website (https://github.com/Minyu96/IBD-and-PC).

## Data availability statement

The original contributions presented in the study are included in the article/**Supplementary Material**. Further inquiries can be directed to the corresponding authors.

## Ethics statement

This research has been conducted using published studies and consortia providing publicly available summary statistics. Besides, no individual-level data was used in this study. Therefore, the ethical approval was waived by the West China Hospital. The patient consent has already been obtained in the primary research of the IIBDGC and PanSan1 program, so they are not required in our study.

## Author contributions

Conceptualization, YP. Data curation, RL and JJ. Formal analysis, JJ and ZW. Funding acquisition, XH and XP. Investigation, ZL, XH, and XP. Methodology, YM, RL, and ZW. Project administration, XH and XP. Software, JJ and XH. Supervision, XH and XP. Validation, ZL, RL, and YP. Visualization, YM and XP. Writing – original draft, YM, ZL, RL, JJ, ZW, YP, and XP. Writing – review & editing, YM, ZL, RL, JJ, ZW, YP, and XH. All authors contributed to the article and approved the submitted version.
